# P01-048 – Systemic onset JIA with coronary artery dilation

**DOI:** 10.1186/1546-0096-11-S1-A51

**Published:** 2013-11-08

**Authors:** N Aktay Ayaz, G Keskindemirci, E Aldemir, N Melikoğlu Kiplapinar, G Aydogan

**Affiliations:** 1 Pediatric Rheumatology, Istanbul Kanuni Sultan Suleyman Education and Research Hospital, Turkey; 2 Pediatric Cardiology, Istanbul Mehmet Akif Ersoy Thoracic and Cardiovascular Surgery Center, Istanbul, Turkey

## Introduction

Systemic-onset juvenile idiopathic arthritis (SoJIA) is characterized with arthritis, fever, typical salmon pink rash, generalized lymphadenopathy and hepatosplenomegaly. The incomplete and atypical presentations of Kawasaki disease (KD) put it to the first order in the differential diagnosis.

## Case Report

A 2-year old boy had been treated as incomplete KD by 2 gr/kg/day of IVIG and 80 mg/kg/day of aspirin at a general pediatrics clinic. From his previous data it was learned that, fever was present for 24 days and it was peaking two to three times a day. He had had a faint rash, arthralgia and lymphadenopathy during follow-up period. But he had’t got conjuctivitis or extremity changes. It was learned that his lips were becoming red when the fever had rised. At that time,he had had very high inflammatory markers. Microbiological work-up had been performed and no etiology to clarify the fever of unknown origin had been found. At his echocardiography, left coronary artery dilatation had been detected and he had been accepted as incomplete KD. But due to unresponsiveness of his complaints and high acute phase response to this treatment protocol, he had been sent to our pediatric rheumatology unit for further diagnostic evaluation. When he was first seen at our outpatient clinic, arthritis of both wrists and knees were noticed. He was internalized and his temperature charts were closely followed. He had two peaks of fever/day with salmon colored rash over his chest. On his physical examination, cervical lymphadenopathy and hepatosplenomegaly were remarkable. There were no peeling of fingers, conjunctivitis and crackles above lips. His hemoglobin was 9.1 gr/dl, leukocyte count was 7600/µl, platelet count was 582000/µl, erythrocyte sedimentation rate was 84 mm/hr, C-reactive protein was 57 mg/l. At his echocardiography z-scores of LMCA was 3.23, LAD was 4.83, RCA was 2.83. At abdominal ultrasound presence of hepatosplenomegaly was confirmed. Bone marrow aspiration was done and exclusion of infiltrating malignant diseases and macrophage activation syndrome were made. The diagnosis was SoJIA with dilatation of coronary arteries. Oral methotrexate of 15 mg/m²/week and pulse steroid of three doses of 30 mg/kg/day with an antiaggregating dose of aspirin were commenced. His fever had subsided and laboratory values began to decline. Both pain and limited range of motion of the affected joints had ameliorated. Even though, still with high z-scores, coronary dilatation began to regress at his follow-up echocardiographic evaluations.

## Discussion

Incomplete and atypical nature of KS at early infancy put forward a diagnostic dilemma. Coronary artery dilatation (CAD) was once accepted as one of the differentiating features of KD and SoJIA. Disease duration and prolonged inflammation may be a causative factor for CAD in SoJIA. According to a previously published study and as in our case CAD might not be an uncommon feature of SoJIA [1]. Pediatricians as the first evaluating physicians should be aware of the symptoms, signs and possibility of coronary artery involvement in SoJIA.

## Disclosure of interest

None declared
